# Assessment of muscle function using hybrid PET/MRI: comparison of ^18^F-FDG PET and T2-weighted MRI for quantifying muscle activation in human subjects

**DOI:** 10.1007/s00259-016-3507-1

**Published:** 2016-09-08

**Authors:** Bryan Haddock, Søren Holm, Jákup M. Poulsen, Lotte H. Enevoldsen, Henrik B. W. Larsson, Andreas Kjær, Charlotte Suetta

**Affiliations:** Department of Clinical Physiology, Nuclear Medicine & PET, Rigshospitalet Glostrup, Copenhagen University Hospital, Ndr. Ringvej 57, DK2600 Glostrup, Denmark

**Keywords:** PET/MRI, Hybrid imaging, Muscle, *T*_2_, Exercise

## Abstract

**Purpose:**

The aim of this study was to determine the relationship between relative glucose uptake and MRI *T*_2_ changes in skeletal muscles following resistance exercise using simultaneous PET/MRI scans.

**Methods:**

Ten young healthy recreationally active men (age 21 – 28 years) were injected with ^18^F-FDG while activating the quadriceps of one leg with repeated knee extension exercises followed by hand-grip exercises for one arm. Immediately following the exercises, the subjects were scanned simultaneously with ^18^F-FDG PET/MRI and muscle groups were evaluated for increases in ^18^F-FDG uptake and MRI *T*_2_ values.

**Results:**

A significant linear correlation between ^18^F-FDG uptake and changes in muscle *T*_2_ (*R* ^2^ = 0.71) was found. for both small and large muscles and in voxel to voxel comparisons. Despite large intersubject differences in muscle recruitment, the linear correlation between ^18^F-FDG uptake and changes in muscle *T*_2_ did not vary among subjects.

**Conclusion:**

This is the first assessment of skeletal muscle activation using hybrid PET/MRI and the first study to demonstrate a high correlation between ^18^F-FDG uptake and changes in muscle *T*_2_ with physical exercise. Accordingly, it seems that changes in muscle *T*_2_ may be used as a surrogate marker for glucose uptake and lead to an improved insight into the metabolic changes that occur with muscle activation. Such knowledge may lead to improved treatment strategies in patients with neuromuscular pathologies such as stroke, spinal cord injuries and muscular dystrophies.

## Introduction

Skeletal muscle tissue accounts for about 40 % of the human body mass and, given its central role in human mobility and metabolic function, any deterioration in its contractile and metabolic properties has a significant effect on human health. Consequently, more attention has been given to the function of human skeletal muscle in an attempt to improve the prognosis and rehabilitation in large patient groups including those with spinal cord injuries [1], muscular dystrophies [2], metabolic dysfunction [3–5], hypertension [6], multiple sclerosis [7, 8] and heart failure [9, 10], and aged individuals [4, 11, 12].

Helping these patient groups requires a detailed understanding of the mechanisms involved in muscle activation and of the specific conditions that must be present for beneficial effects such as neuron growth, angiogenesis and stem cell production to take place. Progress in muscle imaging has to a large degree been due to improvements in PET and MRI that can provide detailed 3D images of cumulative muscle activity throughout the body [13–15]. This spatial information is difficult to obtain using surface electromyography (EMG) [16, 17]. With the recent availability of PET/MRI scanners, it is now possible to obtain PET and MRI measurements simultaneously to fully evaluate the vascular and metabolic processes involved in normal and dysfunctional muscle activity.

Both PET and MRI can produce 3D parametric images, which have been shown to correlate with the intensity and duration of muscle activation [13–15, 18–21]. At present, ^18^F-FDG PET is the gold standard of the two modalities for imaging cumulative skeletal muscle activation since it measures glucose uptake which has been shown to increase with increasing muscle metabolism and activity [13, 14, 22–25]. MRI measurements of muscle activation are based on changes in *T*_2_ values, which is affected by several biological factors including intracellular and intercellular water volumes and acidification [26]. A common assumption is that measured increases in *T*_2_ values in muscle tissue after activation are a result of oedema which has been shown to be the predominant factor causing differences in muscle tissue *T*_2_ values [27, 28]. Although MRI has proven reliable in several studies [15, 21, 26], the lack of clarity as to the mechanisms responsible for the changes in *T*_2_ values remains a hindrance. On the other hand, MRI imaging offers several advantages including its wide range of applications and the fact that it does not use ionizing radiation. MRI also has a higher resolution and anatomic contrast than PET, allowing a more precise analysis of muscle involvement as well as perfusion, fat infiltration and water movement.

The purpose of the present study was, therefore, to use PET/MRI to simultaneously measure ^18^F-FDG uptake and *T*_2_ changes in activated skeletal muscle to determine if MRI can consistently and accurately give 3D measurements of muscle activation equivalent to those with ^18^F-FDG PET. To the best of our knowledge, this is the first study in which such a comparison has been made. A strong linear correlation between *T*_2_ changes and glucose uptake would indicate that MRI could provide a helpful surrogate measurement in metabolic research or a clinical tool for patients with neuromuscular or metabolic dysfunction. The combination of the two modalities could also help meet contemporary challenges in metabolic research and training science such as investigating regulatory mechanisms and improving the effectiveness of training and rehabilitation. On the other hand, discrepancies between the responses measured by the two modalities would open the possibility of extracting complementary information pertaining to muscle activation where different mechanisms are measured with 3D spatial resolution.

## Materials and methods

### Subjects

Ten recreationally active young men (age 24 ± 2 years, BMI 22.9 ± 2 kg/m^2^) volunteered to participate in the present study. None of the subjects had previously participated in systematic resistance training. The Ethics Committee of the Capital Region of Denmark approved the study (protocol no. H-1-2013-146) and the participants gave written informed consent to participate in accordance with the principles of the Declaration of Helsinki.

### Exercise intervention

**Fig 1:**
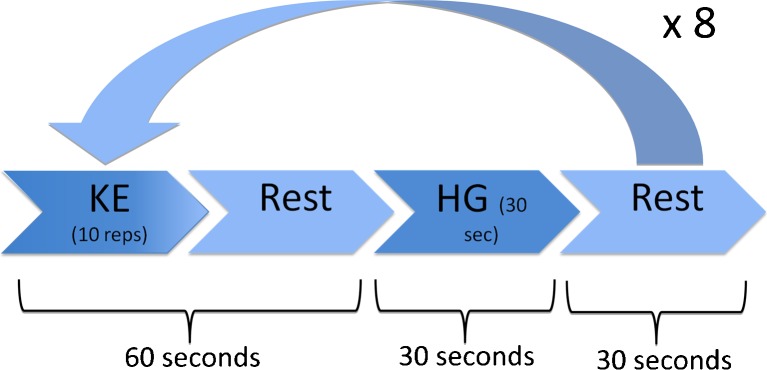
Exercise paradigm. For each set, subjects did ten knee extension (*KE*) repetitions and 30 s of hand-grip (*HG*) exercises with a rest period between such that the set took exactly 2 min. ^18^F-FDG was injected after the KE repetitions in the second set while resting. The subject was moved to the scanner after the HG exercises of the eighth set

The exercise intervention consisted of exercising the right quadriceps and lower left arm muscles of a single limb in order to study both small and large muscle groups, while the same muscle groups of the contralateral limb were at rest. The leg exercise consisted of eight sets of ten knee extension (KE) repetitions with an individually determined repetition maximum load for ten repetitions (10 RM load). Hand-grip (HG) contractions were performed using an Adidas professional grip trainer and subjects were instructed to perform as many contractions as possible in 30 s (Fig. 1). Each exercises was followed by a rest period to produce a repeating KE–rest–HG–rest paradigm of exactly 2 min. Prior to these experiments (1 – 2 weeks), the subjects were familiarized with the KE machine, the seating of the training device (TecnoGym Inc.) was adjusted, and the individual’s 10 RM load was determined for the KE exercise using the right leg only.

### Testing procedure

On the testing day, MRI baseline resting scans were performed prior to exercising. Subjects then performed the eight KE–rest–HG–rest sets of ten KE repetitions and 30 s of HG contractions. After two training sets, 249 ± 3 MBq of ^18^F-FDG was injected, and then the subjects completed the remaining six sets. During exercise great care was taken to ensure that muscles in the left leg and right arm were not used in any way, and thus remained inactive. Accordingly, each individual’s contralateral limb acted as a resting control. Immediately after the exercise intervention, the subject was transported as quickly as possible in an MR compatible wheel chair and positioned in the PET/MRI scanner using the preposition settings from the baseline scan.

### PET/MRI data acquisition

Scanning and reconstruction were performed on a Siemens Biograph mMR PET/MRI scanner (Siemens AG, Erlangen, Germany; software version syngo MR B20P). MR spin-echo and PET dynamic images were acquired simultaneously centred over the mid-femur. Data acquisition was started as soon as possible after exercise depending only on the time required to position the subject in the scanner and acquire a scout scan. *T*_2_ data were acquired using a turbo spin echo sequence with TR 2,500 ms and two interleaved echo times TE_1_ 18 ms and TE_2_ 88 ms. The leg data were acquired first centred over the mid-femur with a 152 × 256 matrix, a pixel size of 1.75 mm, ten transaxial slices of 8 mm and a scan time of approximately 4 min. The arm data were then acquired centred over the maximum cross section diameter of the right arm with eight slices of 8 mm, a 152 × 256 matrix, a pixel size of 1.95 mm and a scan time of 4 min.

^18^F-FDG PET emission data were acquired in list mode simultaneously with the *T*_2_ data using a single bed position for 3 min with the same centring as the *T*_2_ acquisition. PET images were reconstructed using 3D OSEM with three iterations, 21 subsets and 4 mm gaussian postreconstruction filter, using attenuation maps created from MRI Dixon scans. The reconstructed data had an output matrix of 512 × 512 and a resulting voxel size of 1.4 × 1.4 × 2.0 mm which was resized with nearest neighbour interpolation to match the *T*_2_ data for analysis. After ^18^F-FDG PET and MRI *T*_2_ data acquisition, a multibed PET acquisition was performed to visually review the ^18^F-FDG distribution within the body.

### Data analysis

^18^F-FDG PET data and MRI *T*_2_ data are coregistered to ensure identical tissue volumes for region of interest (ROI) and voxel to voxel comparison. *T*_2_ maps were created from two images with echo times of 18 ms (TE_1_) and 88 ms (TE_2_) using the formula $$ {T}_2=\frac{TE2-TE1}{ln\left(\raisebox{1ex}{$TE1\  image$}\!\left/ \!\raisebox{-1ex}{$TE2\  image$}\right.\right)} $$. To reduce the inclusion of blood vessels and fat tissue, a maximum *T*_2_ value of 80 ms and a minimum of 35 ms were used as thresholds for a voxel to be included as muscle tissue. An analysis of muscle groups in the leg and arm was performed using median values from ROIs drawn to include, as much as possible, its entire volume. ROIs drawn in the arm are referred to as either belonging to ‘extensor’ or ‘flexor’ muscle groups. Relative values for both ^18^F-FDG uptake (relGU) and changes in *T*_2_ values (relΔ*T*_2_) were calculated using the equations::

1 $$ relGU = \frac{SU{V}_{muscle}-SU{V}_{ref}}{SU{V}_{ref}},\kern0.5em  rel\varDelta {T}_2 = \frac{{T_2}_{muscle}-{T_2}_{ref}}{{T_2}_{ref}} $$

Here ‘muscle’ refers to the voxel or ROI of muscle and ‘ref’ is the reference muscle tissue ROI. In the arm, the reference muscle tissue was a large ROI including the extensor and flexor muscles of the resting arm. In the leg, the reference tissue ROI was drawn over the vastus muscle groups from the quadriceps of the resting leg. Since the ratio relΔ*T*_2_ normally has small values less than 0.5 as opposed to relGU values, which range from 1 to 7, relΔ*T*_2_ is reported in percent.

### Statistical analysis

The correlation between muscle ROI relΔ*T*_2_ and relGU values was evaluated using linear regression with relGU as the independent variable. In order to use linear regression it is necessary to determine whether the covariance is the same for all muscle tissue independent of the subject or the muscle type. For this reason three ANCOVA analyses were performed to determine if linear regression coefficients were independent of the subject and muscle group, and whether the muscle was from the arm or leg. The first ANCOVA tested for significant differences in regression coefficients when all data was subgrouped by muscle group, the second tested for significant differences between subjects, and the last tested for significant differences between arm and leg data. A similar regression analysis was performed at the voxel level using the mean relΔ*T*_2_ for voxels of muscle tissue grouped by intervals of relGU. Since most MRI kinetic studies link concentration changes with the relaxation rate, *R*_2_ (*R*_2_ = 1/*T*_2_), as opposed to the relaxation time constant, *T*_2_, relΔ*R*_2_ was calculated in a similar manner to the calculation shown in Eq. 1, and the relationship between relΔ*R*_2_ and relGU evaluated by linear regression analysis. Since the exercise paradigm did not include the hamstring leg muscles, these muscle groups were not included in the regression analysis.

Testing for significance was performed using a Student’s *t* test with a threshold *p* value of <0.05. Data are reported as means ± standard deviation (SD). The adjusted coefficient of determination (*R* ^2^) was used to evaluate the goodness of fit of the data to a linear model. All image manipulations, calculations and statistical analyses were performed using scripts created in MATLAB 2013b (MathWorks, Natick, MA).

## Results

**Fig. 2:**
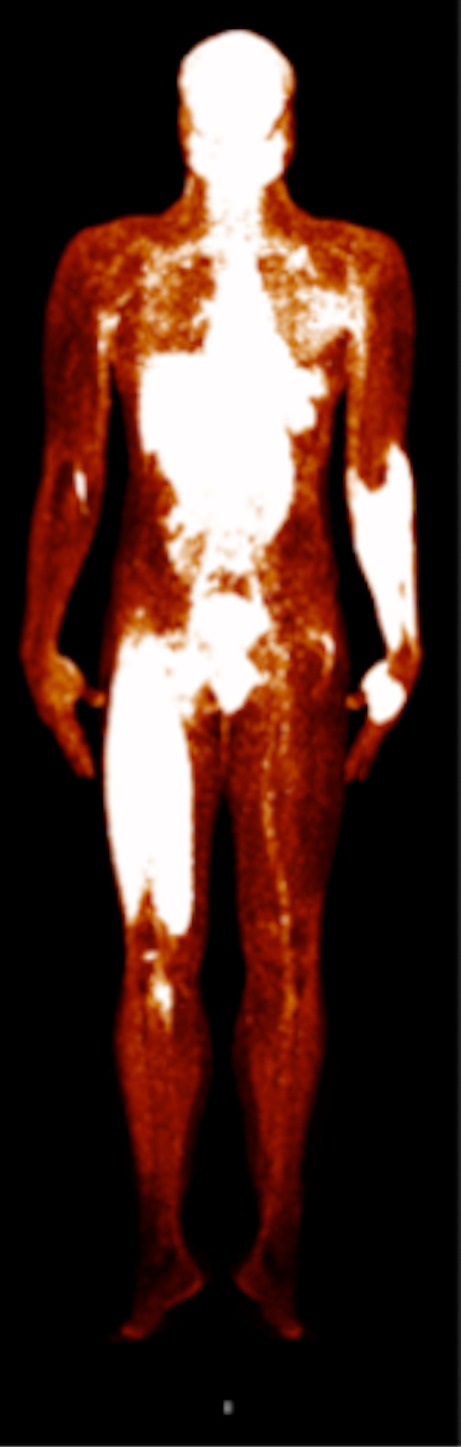
Whole-body ^18^F-FDG PET image of subject after the exercise intervention. The right quadriceps and lower left arm muscles used in the exercise intervention show significantly higher glucose uptake than the resting contralateral limbs

**Fig. 3:**
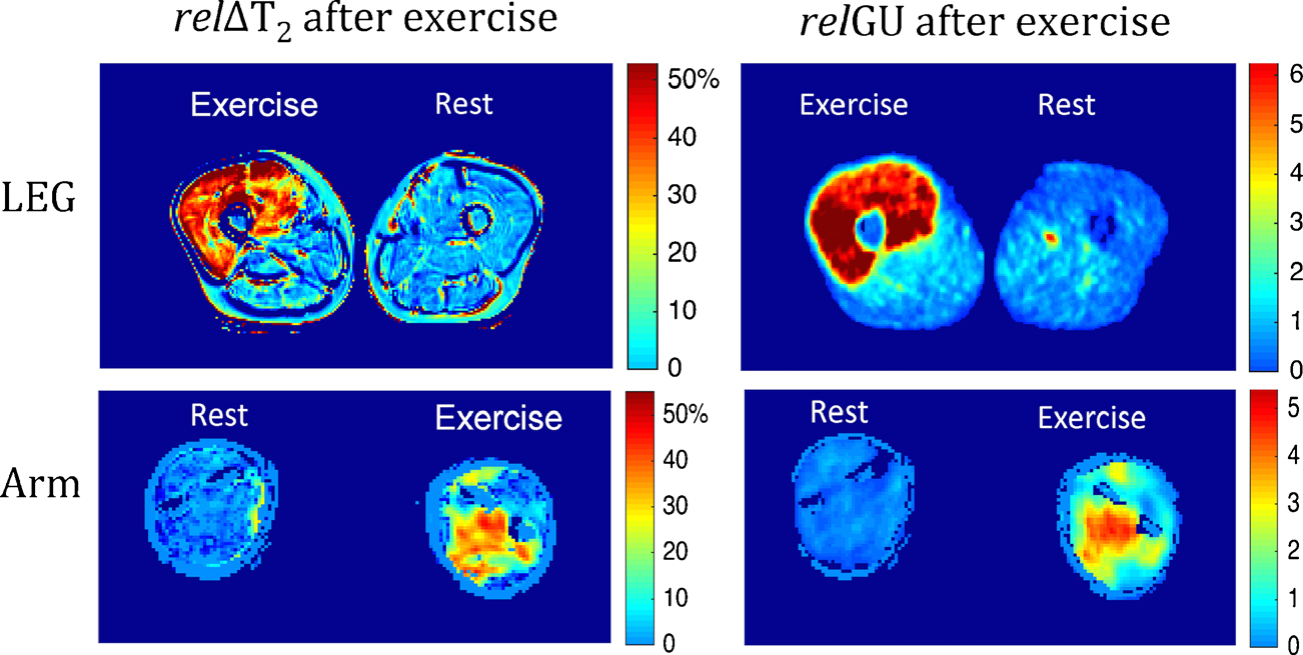
MRI (relΔ*T*_2_) and ^18^F-FDG PET (relGU) images of legs and arms after exercise. *Top*: Cross sections of the mid-femurs in the same subject after unilateral exercise of the quadriceps of the right leg. *Bottom*: Cross sections of the forearms at maximum diameter in the same subject after exercise of the left arm. For both the arms and the legs, the images show the relative increases in *T*_2_ values (relΔ*T*_2_ as precentages) and relative increases in SUV values (relGU as ratios)

**Fig. 4:**
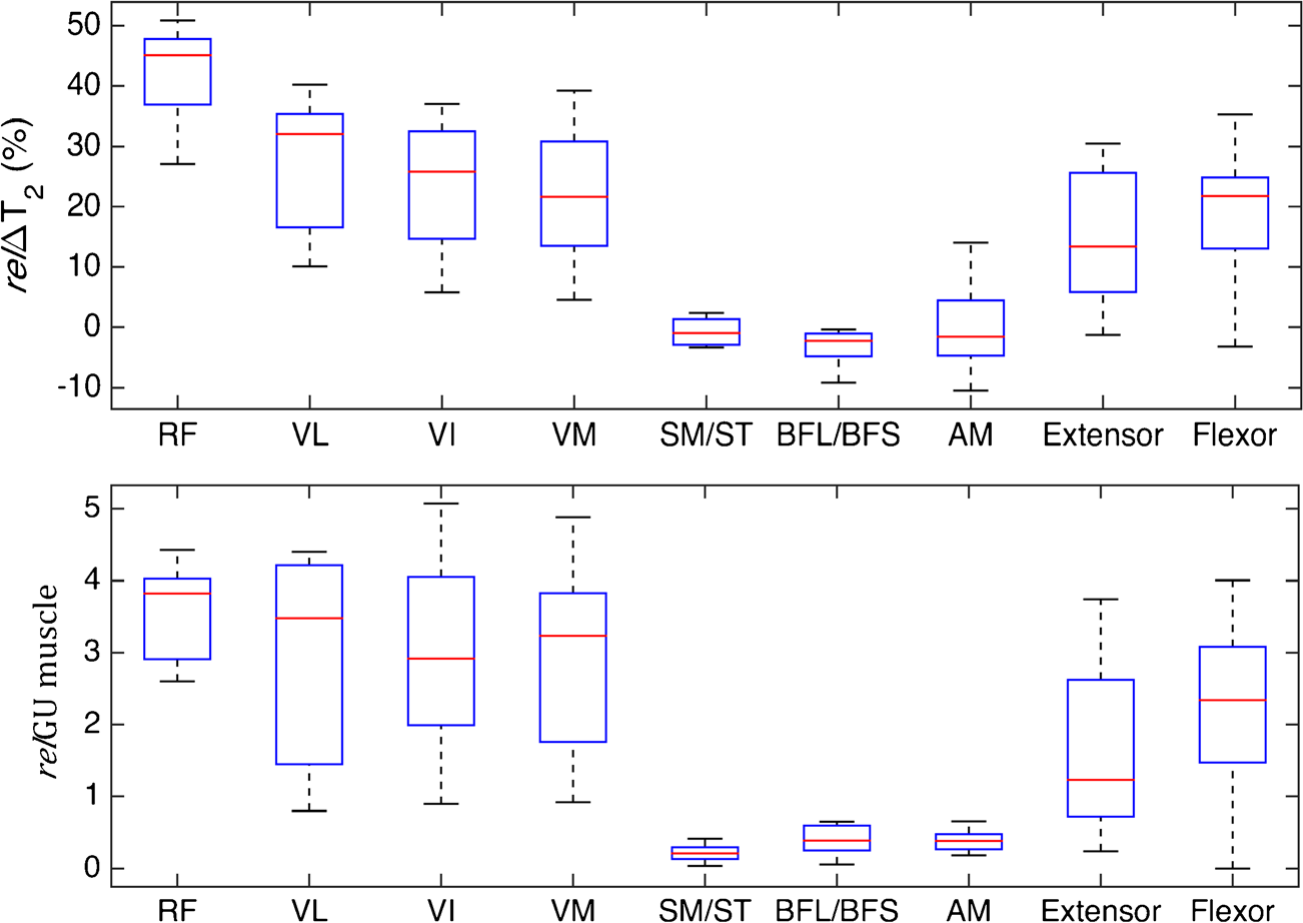
Response of muscle groups to training as measured in terms of relΔ*T*_2_ and relΔGU. The results from nine subjects for each muscle group in exercised arm and leg are shown. Leg quadriceps muscles: *RF* rectus femoris, *VL* vastus lateralis, *VI* vastus intermedius, and *VM* vastus medialus. Leg hamstring muscles: *AM* adductor magnus, *ST* semitendinosus, *SM* semimembranosus, *BFS* biceps femoris short-head, and *BFL* biceps femoris long-head. Lower arm muscle groups: *Extensor* extensor group, and *Flexor* flexor group

**Fig. 5:**
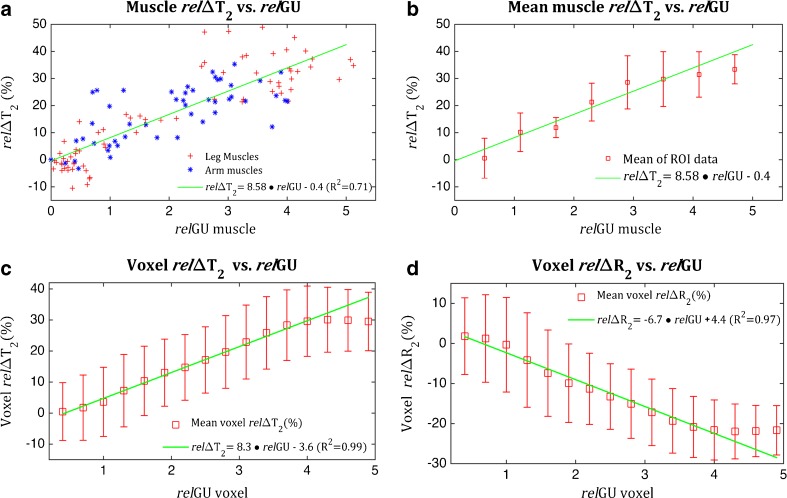
Relationships between relΔ*T*_2_ and relGU in the exercised limbs of all subjects. **a** Values from whole muscle ROIs for all individual muscle groups (as shown in Fig. 3) of exercised arms and legs. **b** The same ROI data as in **a** grouped into the relGU intervals shown and plotted as the mean ROI relΔ*T*_2_ for each interval. **c**, **d** Comparisons including all voxels of muscle tissue showing mean relΔ*T*_2_ (**c**) and mean relΔ*R*_2_ (**d**) for the relGU intervals shown. All *error bars* represent ±standard deviations

All ten subjects completed the training paradigm and the subsequent scanning. One subject’s data were excluded due to technical difficulties during scanning and one subject’s arm data were excluded due to artefacts. The average time from stopping the exercises to starting the ^18^F-FDG PET/MRI scan was 9.1 ± 3.8 min. Both ^18^F-FDG PET and T_2_-weighted MRI scans measured higher intensities in the muscle groups of the exercised quadriceps and exercised arm muscles than in the non-activated muscle groups (Figs. 2, 3 and 4). The spatial distribution of ^18^F-FDG activity and MRI *T*_2_ changes were very similar, even in subjects in whom activation was inhomogeneous throughout the exercised arm or quadriceps muscle (Fig. 4). Activation measurements (relΔ*T*_2_ and relGU) and control *T*_2_ measurements for each muscle group are presented in Table 1. There was a highly significant (*p* < 0.01) linear correlation between the two measurements of activation, relΔ*T*_2_ and relGU, when comparing all exercised arm and leg data on the basis of both whole muscle ROIs and mean voxel values (Fig. 5a, c). The linear regression of muscle ROI relΔ*T*_2_ (%) with relGU as a covariant (*R* ^2^ = 0.71) gave:

**Table 1:** ROI muscle relΔ*T*_2_, relGU and measured *T*_2_ values from nine subjects (means ± standard deviation)

Muscle group		Active limb				Resting limb			
		relΔ*T*_2_ (%)	relGU	*T*_2_ (ms)		relΔ*T*_2_ (%)	relGU	*T*_2_ (ms)	
				Control	After exercise			Control	After exercise
Leg quadriceps muscles	Vastus lateralis	26 ± 10	3.2 ± 1.4	46 ± 2	57 ± 5	0.4 ± 4^a^	−0.1 ± 0.1^a^	48 ± 2	45 ± 3
	Vastus intermedius	24 ± 11	3.1 ± 1.3	45 ± 1	56 ± 5	−1.8 ± 2^a^	0.1 ± 0.0^a^	46 ± 1	44 ± 1
	Vastus medialus	22 ± 11	2.9 ± 1.3	47 ± 3	55 ± 6	3.1 ± 8^a^	0.1 ± 0.1^a^	50 ± 3	47 ± 3
	Rectus femoris	42 ± 8	3.7 ± 0.6	48 ± 4	64 ± 4	23 ± 10	0.0 ± 0.1	58 ± 5	55 ± 4
Leg quadriceps muscles	Semimembranosus/semitendinosus	−1 ± 2	0.2 ± 0.1	47 ± 2	45 ± 2	−2.3 ± 3	0.3 ± 0.1	47 ± 2	44 ± 2
	Biceps femoris long-head/biceps femoris short-head	−3 ± 3	0.4 ± 0.2	45 ± 2	43 ± 2	−1.8 ± 6	0.4 ± 0.1	46 ± 3	45 ± 4
	Adductor magnus	0.1 ± 8	0.4 ± 0.1	47 ± 3	45 ± 4	−2.0 ± 8	0.4 ± 0.2	47 ± 4	45 ± 5
Lower arm muscle groups	Extensor	15 ± 11	1.6 ± 1.0	40 ± 2	46 ± 4	0 ± 4^a^	−0.1 ± 0.1^a^	42 ± 2	43 ± 3
	Flexor	19 ± 10	2.3 ± 1.4	41 ± 2	48 ± 3	0.5 ± 4^a^	0.0 ± 0.1^a^	41 ± 3	44 ± 5

^a^Values calculated using the median of the reference ROI. Therefore, the values for these muscles individually are not zero

2 $$ rel\varDelta {T}_2=8.58\ rel\mathrm{G}\mathrm{U} - 0.4 $$

The ANCOVA analysis showed that the regression parameters (slope and intercept) did not vary significantly between subjects nor between arm and leg muscles. Similarly, there were no significant differences between the muscle subgroups with the exception of the rectus femoris muscle, which had a significantly higher slope of 12. Linear regression of mean voxel relΔ*T*_2_ versus relGU (Fig. 5c) gave a similar slope to Eq. 2, although with an intercept that was significantly lower. The resulting regression equation (*R* ^2^ = 0.99) was:

3 $$ rel\varDelta {T}_2=8.3\ rel\mathrm{G}\mathrm{U} - 3.6 $$

The correlation between mean relΔ*R*_2_ values and glucose uptake (Fig. 5d) was also linear. The regression equation (*R* ^2^ = 0.97) was:

4 $$ rel\varDelta {R}_2=-6.7\ rel\mathrm{G}\mathrm{U}+4.4 $$

In both muscle ROI and mean voxel comparisons, relΔ*T*_2_ values did not increase significantly further for relGU values exceeding a value of 4 (Fig. 5) though only three muscle ROIs were in this range.

Mean muscle *T*_2_ values from control MRI scans obtained prior to exercise were 49.2 ± 3.2 ms in the leg muscle and significantly lower (41 ± 0.8 ms) in the arm muscle (Table 1). There were no significant left–right differences. All muscle groups in the resting leg showed a significant decrease in *T*_2_ values after training with a mean decrease of 5 % (Table 1). The decrease in *T*_2_ values was less pronounced in the hamstring muscle groups than the quadriceps of the resting leg muscles, although not significantly so. The same muscles had positive relGU values (mean 0.4 ± 0.1), which represents a small but significantly elevated glucose uptake. Contrary to resting leg muscles, muscles in the resting arm had higher average *T*_2_ values after training compared to the control scan, although the difference was not significant.

## Discussion

To the best of our knowledge, this is the first simultaneous PET/MRI study of muscle activation. Notably, our ^18^F-FDG PET/MRI data confirms the results of previous MRI studies that have shown increases in muscle *T*_2_ values [15, 20, 21] and PET studies that have shown increases in glucose uptake after muscle activation [13, 14, 18, 19]. More importantly, this study showed a strong linear correlation between relGU and relΔ*T*_2_, which has not been investigated previously to our knowledge. The correlation was found to be consistent when comparing groups of both small and large muscles. Only the rectus femoris muscle had a relΔ*T*_2_/relGU ratio moderately higher than that of other muscle groups. The linear relationship was also consistent for a wide range of measured activities. Despite the controlled exercise paradigm, there was a large variation in the magnitude of measured muscle activities among subjects, muscle groups and even within muscles (Fig. 5a, Table 1). Still, the relGU/relΔ*T*_2_ correlation was found to be consistent regardless of the magnitude of the measured activity even for voxel by voxel comparisons (Fig. 5c). Lastly, this study also showed a decrease in resting leg muscle *T*_2_ values after the subject had exercised the contralateral leg, which has not been reported previously.

The finding of a correlation between an oedema marker (relΔ*T*_2_) and a metabolism marker (relGU) corresponds well with the findings of prior research in muscle physiology and glucose transport [3, 23, 29, 30]. An explanation for the correlation could be that both were blood flow-dependant under the conditions of this study. In a maximal dynamic exercise bout with a small muscle mass, such as the exercise regime in the present study, similar levels of muscle hyperaemia are reached regardless of the arterial blood oxygen content and partial pressure, venous blood pH, femoral vein blood temperature or haemoglobin desaturation [30]. Instead, the immediate vascular response depends on the total workload or, more specifically, the total number of active motor units and the amount of tension they create [23, 29, 31–33]. Thus, the linear correlation between glucose uptake and oedema the linear correlation of each with muscle activation could stem from the linear correlation between blood flow and the degree of muscle activation [29, 30, 33]. MRI and PET measure muscle activity, not by the degree of neural activation or by metabolism itself, but by the associated influx of water and glucose, respectively, and therefore perfusion plays a role in the measurement mechanism of both modalities. Muscular activity cannot, however, be assumed to be homogeneous throughout a given muscle [8, 34]. As also found in previous studies, our results show that even though quadriceps were activated using a strictly controlled paradigm, there was a large variation in the measured activation among subjects, among individual muscle groups and even within a muscle [19–21, 35]. However, the correlation between relΔ*T*_2_ and relGU was found to be consistent in voxel by voxel comparisons and displayed the same inhomogeneous activation patterns.

For an endurance exercise or exercise involving several of the body’s main muscle groups, blood flow would be regulated by many mechanisms [23, 29, 30] and the relΔ*T*_2_/relGU ratios would possibly differ from those found in this study. Most importantly, elevated glucose uptake in active muscle cells could continue even after oedema reaches a maximum. This, we believe, is a plausible explanation as to why increases in the relΔ*T*_2_ values reach a plateau after relGU values exceed a value of around four (Fig. 5). This effect may indicate that muscle activation of longer duration would be more difficult to quantify and the resulting relationship between glucose uptake and oedema would be more complex. The point at which relΔ*T*_2_ reaches a maximum may differ between muscles. Enocson et al. found that during KE exercises MRI changes in the rectus femoris are are greater than those in the vastus muscles at maximum work load [21]. This may explain the slightly elevated relΔ*T*_2_/relGU slope of the rectus femoris muscle shon in the ANCOVA analysis. On the other hand, the relationship remained consistent across a large range of intensities of measured muscle activity and a large range in muscle group size. This indicates that the relΔ*T*_2_/relGU correlation would apply to a variety of training paradigms of shorter endurance.

^18^F-FDG PET and *T*_2_-weighted MRI are increasingly being used to measure muscle activation, but there are still factors that need to be considered. First, it is important to remember that both are a cumulative measurements of muscle activity preceding image acquisition and give little information about how the activity varied over time. The summative time frame includes, though to a lesser degree, the period after exercise as well. Our analysis of ^18^F-FDG PET data assumed, as in previous studies [11, 36], that the levels of radioactivity in plasma are relatively low after the exercise and that the period between the end of exercise and the start of the scan has a minor impact on relative skeletal muscle tissue tracer concentrations. Exercise was stopped 12 min after^18^F-FDG bolus injection and scanning was started on average 20 min injection. During the period from 12 to 20 min after injection, arterial blood activity in an average subject will fall from about 30 % of its maximum to about 20 % [37] which is a relatively small amount, but not negligible. Second, the MR data do not represent muscle tissue during exercise but rather 9 min after exercise. The elevated *T*_2_ values from muscle activation are known to change after stopping exercise and have been found to decay back to preactivation levels in a somewhat exponential fashion with an approximate half-life of 12 min [15]. To date, no accurate model describing the evolution of *T*_2_ values from the end of training to full recovery has been proposed, which means measurements cannot be properly corrected for the time delay.

A third consideration is the choice of reference tissue. The finding that even resting muscle does not have static *T*_2_ values after activation of other muscle groups is a new consideration affecting the accuracy of the calculation of relΔ*T*_2_. Using a control scan prior to exercise so each voxel serves as its own reference to calculate relΔ*T*_2_ would be more representative than defining an ROI in resting muscle as the reference tissue. In this study, using the same reference tissue for calculations of relΔ*T*_2_ and relGU was important in accurately determining the correlation between the two measures and, for this reason, relΔ*T*_2_ is calculated using the same reference ROI as the relGU, instead of using the control scan. Confirming the state of ‘resting’ muscle tissue in calculations of relGU faces the same challenge, and a control scan immediately before exercise begins is not a plausible solution. Several groups have used ROIs drawn over bone marrow as the reference tissue or preferably blood samples to alleviate this problem when using ^18^F-FDG PET [36]. In the present study, the inactivity of resting muscles used as the reference tissue was controlled using *T*_2_-weighted MRI data from before to after exercise. However, standardization of reference measurements for the calculation of relGU may give more accurate quantification of the correlation between oedema and relGU for comparison between subjects.

The strengths of ^18^F-FDG PET and MRI are not just the possibility for subjects to move freely during exercise, but that the resulting activity can be measured throughout the body, including deeper muscles, and mapped in 3D with high resolution. Traditionally, muscle activation has been measured with EMG which provides good temporal resolution during exercise but has a limited ability to differentiate between muscle groups and cannot easily measure deep muscles [17]. Given the inhomogeneity of activity between muscles and within a muscle group, surface measurements alone provide information of less value than 3D mapping. In studies in which it is important to reveal changes in magnitude or strategy over time, and which therefore require EMG, images acquired with PET or MRI would be able to supply complementary spatial information revealing intramuscular and intermuscular heterogeneity. The wide range of applications of MRI also allows mapping of muscle to be combined with other MRI measurements of interest such as spectroscopy, diffusion-weighted imaging and blood oxygen level-dependent imaging.

Given that skeletal muscle tissue plays a major role in metabolic regulation [3–5], it is important to be able to evaluate in vivo skeletal muscle activation and metabolism and thereby increase our understanding of musculoskeletal function. The results of the present study contribute to this growing field of metabolic research by characterizing skeletal muscle tissue using the correlation between oedema (relΔ*T*_2_) and metabolism (relGU) during its activation. The high correlation between the two parameters provides a new tool for analysing muscle activation allowing MRI to be used as a surrogate measure in a broad range of research areas such as sports performance, and the effects of lifestyle, ageing and rehabilitation. This approach also allows PET/MRI to be used as a diagnostic tool to differentiate muscle groups in patients having a relΔ*T*_2_/relGU correlation that differs from that of healthy subjects.

### Conclusion

This is the first PET/MRI study of muscle activation. Analysis of the data showed a significant linear correlation between ^18^F-FDG uptake and changes in muscle *T*_2_ in groups of both small and large muscles. Accordingly, it seems that changes in muscle *T*_2_ may be used as a surrogate marker for glucose uptake.
